# Supplementary material for: Splice-Junction-Based Mapping of Alternative Isoforms in the Human Proteome
Source: Cell Rep. Author manuscript; Available in PMC 2020 Jan 15. (PMC6961840; doi:10.1016/j.celrep.2019.11.026)
Supplement: 3 [file NIHMS1546469-supplement-3.zip › DF2/PXD006675/TricuspidValve-39-O43795-EAVTTIAAYWHGTQVR.pdf]

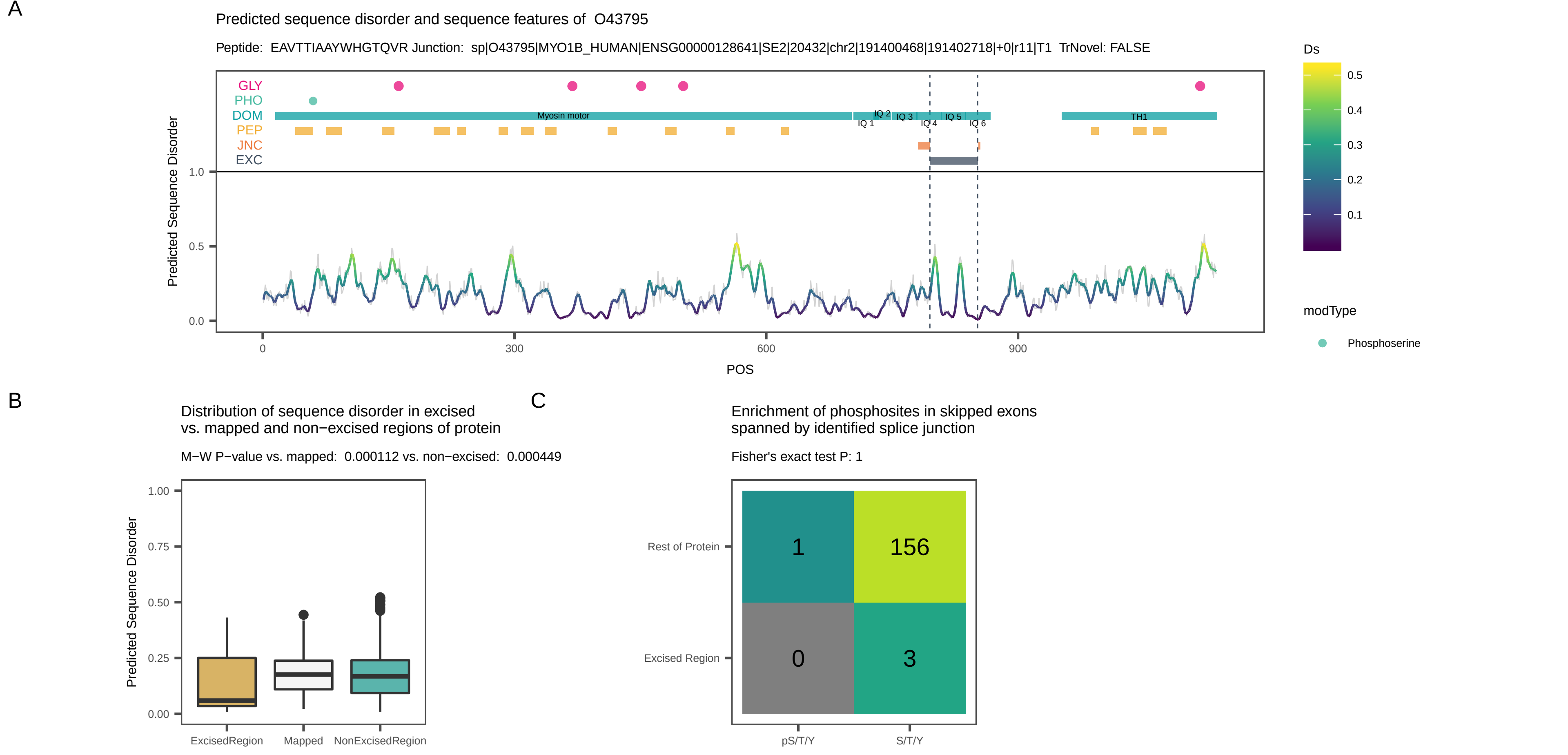
A
Predicted sequence disorder and sequence features of O43795
Peptide: EAVTTIAAYWHGTQVR Junction: sp|O43795|MYO1B_HUMAN|ENSG00000128641|SE2|20432|chr2|191400468|191402718|+0|r11|T1 TrNovel: FALSE
GLY
PHO
DOM
PEP
JNC
EXC
Myosin motor
IQ 1
IQ 2
IQ 3
IQ 4
IQ 5
IQ 6
TH1
Predicted Sequence Disorder
1.0
0.5
0.0
0
300
600
900
POS
Ds
0.5
0.4
0.3
0.2
0.1
modType
Phosphoserine
B
Distribution of sequence disorder in excised
vs. mapped and non−excised regions of protein
M−W P−value vs. mapped: 0.000112 vs. non−excised: 0.000449
Predicted Sequence Disorder
1.00
0.75
0.50
0.25
0.00
ExcisedRegion
Mapped
NonExcisedRegion
C
Enrichment of phosphosites in skipped exons
spanned by identified splice junction
Fisher's exact test P: 1
Rest of Protein
Excised Region
1
156
0
3
pS/T/Y
S/T/Y
